# Development of a scale to measure individuals’ ratings of peace

**DOI:** 10.1186/1752-1505-8-17

**Published:** 2014-09-27

**Authors:** Howard Zucker, Roy Ahn, Samuel Justin Sinclair, Mark Blais, Brett D Nelson, Thomas F Burke

**Affiliations:** 1 Division of Global Health and Human Rights, Department of Emergency Medicine, Massachusetts General Hospital, Boston, MA, USA; 2 Harvard Medical School, Boston, MA, USA; 3 Department of Psychiatry, Massachusetts General Hospital, Boston, MA, USA

**Keywords:** Metrics, Global health, Developing world, Conflict zones, Peace-building, Scale

## Abstract

**Background:**

The evolving concept of peace-building and the interplay between peace and health is examined in many venues, including at the World Health Assembly. However, without a metric to determine effectiveness of intervention programs all efforts are prone to subjective assessment. This paper develops a psychometric index that lays the foundation for measuring community peace stemming from intervention programs.

**Methods:**

After developing a working definition of ‘peace’ and delineating a Peace Evaluation Across Cultures and Environments (PEACE) scale with seven constructs comprised of 71 items, a beta version of the index was pilot-tested. Two hundred and fifty subjects in three sites in the U.S. were studied using a five-point Likert scale to evaluate the psychometric functioning of the PEACE scale. Known groups validation was performed using the SOS-10. In addition, test-retest reliability was performed on 20 subjects.

**Results:**

The preliminary data demonstrated that the scale has acceptable psychometric properties for measuring an individual’s level of peacefulness. The study also provides reliability and validity data for the scale. The data demonstrated internal consistency, correlation between data and psychological well-being, and test-retest reliability.

**Conclusions:**

The PEACE scale may serve as a novel assessment tool in the health sector and be valuable in monitoring and evaluating the peace-building impact of health initiatives in conflict-affected regions.

## Background

The evolving concept of peace-building has crossed multiple disciplines. Whether involving health or security, agriculture or technology, efforts have been made to foster the development of peace through creative programs. For instance, peace theorist Johan Galtung proposed a “triangular syndrome of peace in which cultural peace engenders structural peace, with symbiotic, equitable relations among diverse partners, and direct peace with acts of cooperation, friendliness and love [1].” In the 1980s initiatives involving non-governmental organizations focused on the interplay between peace and health. The World Health Organization’s ‘Health as a Bridge to Peace’ program [2] serves as an example of efforts to acknowledge and cultivate the peace-health interface. Advocates believe that providing primary care health services can improve peace and promote security within a conflict-ridden region [3]. The World Peace Through Technology Organization (WPTTO) advocates the use of technology to inspire peace and “facilitate the evolution of community development between global citizens.” Led by the United Nations’ Institute for Advanced Studies, the Agriculture for Peace project examines the interface between social science and agriculture utilizing the themes of governance, globalization, environment, and security. Despite such innovative ideas there is no gold-standard measurement tool to determine effectiveness of such programs in improving peace within an individual or community. In fact, criticism has been waged in this regard [4]. With the absence of psychometrically sound and well-defined tools to capture the utility of any program, the concept of creating peace within society remains vague.

The topic of peace measurement tool has received considerable attention in the field of international affairs. Much of the literature emphasizes the need for development of reliable metrics of progress, and impact, related to peace-building initiatives. Bush provides a measurement framework for evaluating “development projects in conflict zones [5].” In addition, he proposes sample performance indicators at multiple levels—examples include “perceptions of individual and collective security” and “level of tolerance to cultural or political differences” at the individual level, and “political representation” and “level of economic or employment discrimination” at the macro-social level [5]. Other commentators provide organizations guidance on how to develop their own evaluation systems, namely centered on “logic model” development. The Kroc Institute for International Peace Studies’ *Reflective Peacebuilding Toolkit* devotes chapters to helping organizations generate theories of change around their work as well as the corresponding output and outcome indicators [6]. Researchers at the University of New South Wales developed its Peacebuilding Filter as a practical alternative to the logic model [7]. Similarly, a United States Institute of Peace “Stabilization and Reconstruction” publication describes the need for projects to discern *outputs* from *outcomes*—the latter requiring primacy in evaluation—and provides practical advice on the process of developing indicators (e.g., “Depoliticize metrics,” “Create buy-in from leaders and staff”) [8]. Beyond these practitioner-focused publications, the new Global Peace Index sets out to compare *countries* on multiple indicators of peace (e.g., Gini coefficient of income inequality) [9]. Relatedly, Almedom calls for the development of a “multidimensional resilience index” to “compile important information about how people actually cope with emergencies instead of focusing only on their vulnerabilities to the adverse impacts.” In Almedom’s conception, a “resilience index” would comprise social capital, sense of coherence, and related extant psychological concepts [10].

However, peace measurement has not been well-explored in the field of global public health. To our knowledge, no biomarkers have been identified to measure peace, and no studies have reported the development of validated and reliable psychometric measures related to the experience of peace. Consequently, researchers have yet to directly address the question, “Can health interventions create a sense of peace among individuals and communities?” In the absence of such measures, this study developed and validated a new peace measurement tool.

## Methods

This paper reports on the development, pilot testing, and initial validation of the Peace Evaluation Across Cultures and Environments (the PEACE scale), a psychometric tool designed to assess an individuals’ experience of peace across multiple, related psycho-social domains (e.g., emotional distress, security, safety, social cohesion, access to basic necessities). As a multidimensional measurement tool, the scale measures how individuals rate their own sense of ‘peace.’ The process utilized for development of the pilot PEACE tool was both systematic and multidisciplinary, and informed in part by theoretical frameworks from the research psychology and health fields. The study received ethical review and approval from the Institutional Review Board of Partners Healthcare (Boston, MA, USA).

The study authors convened an initial meeting of 13 discussants in Boston, MA, on October 29, 2009, to start developing a working definition of peace. Discussants, which included five of the study authors, were selected based on their professional experience in psychology, psychiatry, global health/medicine, human rights, and/or international development. The meeting was recorded and subsequently transcribed by a research assistant. Informed by the issues raised in this meeting as well as a review of the academic literature on peace, the study authors developed a working definition of “peace” (“*A feeling of calm and/or freedom from struggles within self and others in a non-violent environment where hope outweighs resignation*”) and identified concepts or themes associated with the experience of peace. Most definitions were broad and focused on the absence of negative conditions, such as violence and hatred, and the presence of positive conditions such as harmony and connectivity [11]. Further structured, in-person discussions of the study authors resulted in a list of core constructs underlying Peace. These lists were collated and organized into domains. These domains included the following seven constructs: emotional tone/sense of calm, agency or locus of control, hope/optimism; tolerance of others; access to basic necessities, personal safety/absence of violence, and a sense of group or social connectedness.

Given the overall conceptualization, an initial item pool (approximately 10 items per domain) was written to represent each of the underlying seven constructs. The initial item pool was then subjected to expert review, and all items were rated in terms of clarity, face validity, and responsiveness to change. Items were then re-drafted based on feedback, and a beta version of the tool was developed. Based on this process, a 71-item beta version of the Scale was created for initial fielding. Items were presented in a semi-random order over the Scale, and each item was rated on a five-point Likert-type response Scale (Not at all true, A little true, Moderately true, Quite a bit true, and Completely true). Advantages to using a five-point magnitude scale over dichotomous (e.g., Yes/No) or over scales with fewer categories include improving overall reliability and, ultimately, the ability to demonstrate validity.

During the initial fielding both the PEACE scale-Beta Version (PS-BV) and the Schwartz Outcome Scale were administered to participants. The Schwartz Outcome Scale-10 [12] is a brief 10-item tool that measures psychological health and distress [13]. Higher SOS-10 scores indicate greater psychological health while lower scores indicate increased emotional distress [14,15]. The SOS-10’s psychometric properties and construct validity are well established [15].

The psychometric functioning of the PEACE scale was evaluated on a sample of 250 adults recruited through three sites in the United States: the emergency department of a large northeastern teaching hospital, a northeastern community center, and a northeastern university. We obtained permission from these study sites to hand out questionnaires (i.e., waiting room of the hospital emergency department, the lobby of the community center, and inside a library of the university). The sample had a mean age of 42 years (standard deviation [SD] = 19 years) and was 51% female. Research assistants recruited subjects until a target number of subjects were enrolled at each of the three sites. The majority of participants were high school graduates (70%) and Caucasian (64%). After being informed of the study and providing consent, the participants were handed the study materials and asked to complete the forms.

The PEACE instrument was initially evaluated in terms of several core underlying psychometric assumptions. First, adjusted item-to-scale correlations were examined to ensure that all items were strong, linear measures of their intended construct, with the item removed to correct for overlap. Second, items were also evaluated to ensure that they were stronger measures of their parent construct, as opposed to other competing constructs/scales in the model. This assumption was evaluated using a Steiger’s t-test for dependent correlations. Third, internal consistency reliability was evaluated using Cronbach coefficient alpha for each of the scales to ensure scale reliability. However, given that reliability is contingent on the number of items present and the scales varied in number of items, a second estimated Cronbach’s alpha was calculated using the Spearman Brown formula, assuming a 10-item scale for purposes of more direct comparisons between scales. Fourth, scale-level correlations were evaluated relative to each scale’s reliability to ensure that each scale contributed unique variance to the model. In cases where reliability is low and scale-level correlations are high, this assumption is not met. Fifth, a principal axis factor analysis (PAF) was employed to determine the higher-order latent structure of the Peace Scales. Finally, construct validity of the PEACE scales were evaluated by examining mean differences across high and low “well-being” groups based on the Schwartz Outcome Scale (SOS-10) – a well validated measure of psychological well-being.

## Results

### Item analyses and scale refinement

The sample size of the present study was not sufficient to employ an item-level factor analysis as a means of assessing the dimensionality of the item pool (71 items and approximately 216 subjects with complete data). Therefore, item adequacy (i.e., degree to which items were associated with their hypothesized scales) was assessed using internal consistency (coefficient alpha) and by reviewing the pattern of convergent and divergent item-to-scale correlations. To do this, the items were organized into their intended Scales, and convergent and divergent correlations were obtained. Adjusted item-to-scale correlations were used to assess item convergence (the degree to which items were associated with their target Scale). Divergent correlations, the correlation of an item with the six non-target scales, were used to assess item discrimination. To be retained on a Scale, each item had to demonstrate an adjusted item-to-scale correlation ≥0.30 [16] and have no divergent (off-scale correlations) equal to or greater than its adjusted item-to-scale correlation. Applying these criteria the initial item pool was reduced from 71 to 41 items. Table 1 shows the results of the item-level analysis for each PEACE scale. As Table 1 shows, the PEACE scales generally had acceptable internal consistency despite having only six items per scale (expect for Basic Needs, which was composed of only five items). Next, we applied the Spearman-Brown Prophecy formula to estimate the internal consistency of each PEACE scale if it contained 10 items with these items at the current level of inter-correlation. Results from the Spearman-Brown Prophecy formula revealed that all but one scale (Tolerance) would achieve the acceptable level of 0.80, suggested by Nunnally & Bernstein [16], given their current psychometric properties but expanded to 10 items.

**Table 1:** PEACE scale item level analyses

**PEACE scales**	**Items**	**Mean/SD**	**Alpha**	Alpha estimated^*^	**Mean Inter-item r**
Emotional tone	6	21.9/4.7	0.83	0.88	0.45
Agency^#^	6	12.2/3.9	0.72	0.80	0.31
Hope^#^	6	11.1/3.9	0.73	0.81	0.33
Tolerance^#^	6	9.7/3.3	0.65	0.74	0.25
Basic Needs	5	22.4/3.2	0.69	0.82	0.31
Safety	6	24.4/4.3	0.79	0.85	0.38
Group Cohesion	6	23.7/4.4	0.76	0.83	0.35

Note. N’s vary between 210 and 225 based on missing data. *Alpha estimated using the Spearman-Brown formula for 10-items per Scale. ^#^Lower scores reflect high levels of this domain.

### Correlation analyses

Correlations were computed for PEACE scales (scale inter-correlation) and the SOS-10 (concurrent validity). Table 2 is organized with the observed reliability (internal consistency) for the PEACE scales in the off-diagonals followed by the across scale inter-correlations. Together these data provide an additional assessment of scale convergence as it is expected that for each scale the reliability correlation will exceed the off-scale correlations (inter-correlations). A review of Table 2 shows that each PEACE scale demonstrated adequate convergence as their reliabilities exceeded the off-scale correlations. In addition, Table 2 suggests that the PEACE scales are only moderately inter-correlated. Table 2 shows that all PEACE scales were significantly correlated with the psychological well-being (SOS-10 scores). The strongest correlations with the SOS-10 were obtained for the Emotional Tone, Agency, and Hope Scales. While somewhat smaller in magnitude, statistically significant correlations were also noted between the SOS-10 and Basic Needs, Safety, and Group Cohesion Scales. These correlations provide some initial support for the concurrent validity of the PEACE scales. Given that the PEACE scales showed only mild-to-moderate inter-correlation, we next employed factor analysis to assess the latent structure of the PEACE scales.

**Table 2:** PEACE scale analyses: reliabilities, inter-correlations, and current validity correlations

**Scales**	**ET**	**Agency**	**Hope**	**Tolerance**	**BN**	**Safety**	**GC**	**SOS**
ET	(0.83)							
Agency	−0.57	(0.72)						
Hope	−0.56	0.57	(0.73)					
Tolerance	−0.36	0.35	0.35	(0.65)				
BN	0.25	−0.44	−0.33	−0.16	(0.69)			
Safety	0.40	−0.41	−0.40	−0.17	0.48	(0.79)		
GC	0.31	−0.45	−0.35	−0.25	0.20	0.39	(0.76)	
SOS	0.58	−0.50	−0.51	−0.29	0.18	0.35	0.30	(0.89)

Note. N’s vary from 208 to 222. ET = Emotional Tone, BN = Basic Needs, GC = Group Cohesion and SOS = Schwartz Outcome Scale. Scale reliability (internal consistency) is in the off diagonals. All correlations are significant at p < 0.01.

### Factor structure of PEACE scales

A principal axis factor analysis (PAF) was employed to determine the higher-order latent structure of the PEACE scales. The PAF revealed two factors with eigenvalues greater than 1.0. These two factors were extracted and obliquely rotated using a Promax rotation, given the hypothesized relationship between the overall factors (Table 3). The two-factor solution contained meaningful (0.35 or greater) loadings for the seven PEACE scales and accounted for just over 60% of the variance in the correlation matrix. The two factors were moderately inter-correlated (0.63). The obtained factor structure suggests that the PEACE scale may measure two broad but related domains: one concerned with the psychological (Emotional Tone, Agency, Hope, and Tolerance) aspects of peace and the other focused on social/environmental (Basic Needs, Safety, and Group Cohesion) aspects of peace.

**Table 3:** PEACE scale (pattern matrix) factor structure with Promax rotation

**PEACE scales**	**F-1**	**F-2**	**h**^**2**^
Emotional tone	67		62
Agency	−75		55
Hope	70		53
Tolerance	54		23
Basic needs		50	30
Safety		88	65
Group Cohesion		35	30
% Variance	45	15	

N = 212. Factor loadings and commonalities (h^2^) are presented without decimal points. Only loadings greater then 30 are presented.

### Known group validation

Using the SOS-10 and education level as criterion measures, we conducted two preliminary known groups validation (i.e., discriminative validation) analyses [17]. In the first analysis, the impact of psychological well-being on the PEACE scales was explored by splitting the sample at the mean SOS-10 score (48). Splitting the sample in this manner essentially created groups of high and low well-being subjects. Independent t-tests were conducted to explore between-group differences across the PEACE scales. Based upon this mean (SOS) score split, 126 subjects were assigned to the high well-being group and 90 subjects were placed in the low well-being group. Table 4 shows that the two groups differed significantly across all seven PEACE scales. In addition, the effect sizes (Cohen’s *d*[18]) for these differences were all within the medium-to-large effect range (0.65 to 1.2).

**Table 4:** High and low well-being groups, t-tests, and effect size

**Peace**	**Above mean**	**Below mean**	**t**	**p**	***d***
**Scales**	**Mean/SD**	**Mean/SD**			
Emotional tone	23.9/3.6	19.0/4.5	8.8	<0.000	1.2
Agency	10.5/3.2*	14.6/3.5	8.8	<0.000	1.2
Hope	9.5/3.0*	13.3 4.0	8.0	<0.000	1.0
Tolerance	8.8/2.8*	11.02/3.5	4.9	<0.000	0.7
Basic needs	23.0/2.8	21.5/3.5	3.3	<0.001	0.65
Safety	25.75/3.7	22.9/4.4	5.5	<0.000	0.70
Group cohesion	24.9/4.1	22.0/4.2	5.0	<0.000	0.70

Note. *indicates lower scores are in the healthy direction. *d* = Cohen’s *d* (effect size).

For the second discriminative analysis, the sample was divided based on education level (high school graduates and non-high school graduates). There were 147 subjects with at least a high school education and 60 subjects with less than a high school education. Again, independent t-tests were used to assess between-group differences across the PEACE scales. Table 5 shows that only the PEACE scales associated with social/environmental aspects of peace (Safety, Basic Needs, and Group Cohesion) were significantly different across these two groups. The effect sizes for these differences were small-to-medium effect ranges (0.32 to 0.48).

**Table 5:** Education level group split with t-tests and effect size

**Peace**	**High school and above**	**Less than high school**	**t**	**p**	***d***
**Scales**	**Mean/SD**	**Mean/SD**			
Emotional tone	22.3/4.2	21.3/5.7	1.1	NS	
Agency	11.9/3.5	12.5/4.5	1.4	NS	
Hope	10.8/3.8	11.7/4.4	1.4	NS	
Tolerance	9.7/3.1	9.6/3.6	0.31	NS	
Basic needs	22.8/2.7	21.3/4.2	3.0	<0.01	0.47
Safety	25.0/4.0	23.0/4.7	3.1	<0.01	0.48
Group cohesion	24.1/3.8	22.7/5.4	2.1	<0.05	0.32

*d* = Cohen’s *d* (effect size).

### Test-retest reliability

Test-retest data were available for 20 subjects who completed the PEACE scale approximately two weeks apart. For these subjects the test-retest reliability for the total PEACE scales (all items) was 0.89 (P < 0.001). The retest reliabilities correlations (r_tt_) for the Peace Subscales were as follows: Emotional Tone = 0.85, Agency = 0.80, Hope = 0.81, Tolerance = 0.82, Basic Needs = 0.78, Safety = 0.90, and Group Cohesion = 0.82. All retest correlations for the subscales were statistically significant (p < 0.001). In addition, the total score for the PEACE scale was stable over the retest interval; the initial mean score was 223.44 (SD = 9.85), and the retest mean score was 225.05 (SD = 9.90), with the difference being non-significant (t, [18] = −1.15, p = 0.15). These data indicate that the PEACE scale and its subscales have strong test-retest reliability and good total score stability.

## Discussion

This pilot study demonstrates that our PEACE scale has acceptable psychometric properties for measuring an individual’s level of peacefulness or sense of peace. The study also provides initial reliability and validity data for the PEACE scale, and presents a potential two-factor higher order structure of the PEACE scale.

The study is limited by the small number of participants in our sample and the fact that these were convenience samples at the three study sites. Also, this study did not attempt to test study responsiveness (i.e., sensitivity to change) of the scale. Finally, this study did not conduct interviews with people experiencing post-conflict interventions during the scale development phase. Omitting this significant group from the qualitative phase of instrument development risks missing important aspects of the conceptual basis of the instrument, and may thus compromise its content validity. However, this population could not be included due to resource constraints. Nonetheless, this scale contributes a new scale to an emerging area of global health that is under-developed. By analogy, in the 1980s, the World Health Organization began developing a model of functioning, which highlights the continuum between biological/cellular processes going on within the person and the environment in which they live. Although the International Classification of Functioning (ICF) was developed to model variance in health and functioning, it provides a paradigm for conceptualizing individual and community peace – as it frames the process in terms of the complex and dynamic relationship between the person and his/her environment. For example, someone with a complex set of health conditions and/or disabilities may still be able to engage in life in a meaningful way depending in part on the resources available to them in the environment. Conversely, a person who is healthier, relatively speaking, may be much more limited if the environment has no resources. The infinite shades of grey between these levels reflect this complex interaction. The ICF was ultimately supported by all 191 World Health Organization Member States in 2001 and has received global endorsement.

To date, little has been done to examine or measure Peace at the level of the person and immediate environment. The PEACE scale fills this void and allows for more fine-grain analysis of peace creation. The availability of such a measure helps supplement and enhance the value of information obtained from more global measures like the GPI. Table 6 presents an initial organization of the PEACE tool and the ways in which these constructs map onto the ICF, as well as how the PEACE tool complements existing measurement frameworks (e.g., GPI).

**Table 6:** Developing a measurement model for PEACE

**Continuum**	**World Health Organization ICF Conceptual Framework**	**Measurement domains identified in focus groups**	**Proposed measurement system/method**
Individual	Body functions/structures and physical functioning	1. Bio-markers/physical health	Biological assessments of physical functioning
Interpersonal/social	Participation/engagement in the community	2. Emotional tone/sense of calm	Development of a psychometric (PEACE) test instrument
		3. Agency/sense of control	
		4. Hope/optimism for the future	
		5. Tolerance	
		6. Access to necessities	
		7. Safety/absence of violence	
		8. Group connectedness	
National/global	Environmental factors	Global Peace Index (GPI) would occur at this level	

Our PEACE scale may have many applications in the field of international peace-building, and is wholly consistent with the mandate of United States Institute of Peace “to help prevent and resolve violent conflicts, promote post-conflict stability and development, and increase peace-building capacity, tools, and intellectual capital worldwide [19].” Despite trillions of dollars spent globally on waging war and securing peace, there is a dearth of scientifically-valid measurement of peace at the individual as well as community level. As a result, society subjectively assesses the impact of conflict. Whether involving past tragedies in Rwanda, Darfur, or other fragile regions including Egypt, Syria, and Libya, our PEACE tool could be valuable in the evaluation of purported peace-generating interventions, especially health interventions.

By translating and validating this instrument across geographic, political (e.g., conflict, post-conflict zones), and cultural contexts around the world, the tool will be a new, robust scientific tool to measure peace. A carefully designed psychometric scale will provide a reference point when examining peace-related interventions in a community (e.g., health programs) and help develop new ways of understanding the impact of interventions on individual and community peace. For example, a scale could be used in program evaluations to assess changes in ‘peace’ scores, i.e., pre- and post-program intervention.

## Conclusions

Applications of this valid and reliable community peace index will generate insights on how peace can be measured, accounted for, taken away, and even created. We believe it lays the foundation for an entirely new field of study. By capturing the essence of stability and social capital in a community, it is possible to tailor interventions to achieve the maximum benefit in terms of optimizing peace. As a well-regarded and scientifically proven measuring tool with psychometric utility, the PEACE tool can be adjusted and applied to virtually any setting – from local confined environments (e.g., prisons) to communities emerging from civil conflict.

Efforts generated in the global health sphere can incorporate the PEACE tool. The President’s Emergency Program for AIDS Relief (PEPFAR) has reached 60 million people [20]. Measuring community peace before and after implementation of PEPFAR programs may contribute to estimating the effectiveness of such interventions. In a similar vein, the microcredit summit campaign led by Bangladesh’s Grameen Bank has provided grants to over 130 million clients [21]. The impact of microfinancing on peace through this tool is likely to be enormous. Global estimates suggest a third of all women have been beaten or forced into sex in their lifetime [22]. Public and private efforts to eliminate such atrocities may help bring peace to a community in peril. The Peace tool could evaluate the effectiveness of such interventions. The PEACE tool may benefit teams working within the United Nations as well as leaders in State and Defense Departments across the globe. As one begins to study the effectiveness of interventions, the knowledge accrued would have relevance to many organizations working in all social sectors, including, but not exclusively, the health sector.

## Abbreviations

GPI: Global peace index; PAF: Principal axis factor analysis; PEACE: Peace evaluation across cultures and environments; PEPFAR: President’s emergency program for AIDS relief; SD: Standard deviation; WPTTO: World peace through technology organization.

## Competing interests

The authors declare that they have no competing interests.

## Authors’ contributions

HZ, RA, SJS, MB, BDN, and TFB conceived of and designed the study. BN, RA, TFB led the focus group studies. HZ, RA, and TFB led the data collection. SJS and MB carried out the scale analysis and interpretation. HZ drafted the initial manuscript. All authors read and approved the final manuscript.
